# Draft genome sequence of *Aspergillus ochraceus* VKM-F4104D (L-1) producing peptidase activator of protein C

**DOI:** 10.1128/mra.00416-24

**Published:** 2024-09-30

**Authors:** Anna Shestakova, Daria Surkova, Artem Fatkulin, Alexander Osmolovskiy, Elizaveta Popova

**Affiliations:** 1 Faculty of Biology, Department of Microbiology, M.V. Lomonosov Moscow State University, Moscow, Russia; 2 Faculty of Biology and Biotechnology, HSE University, Moscow, Russia; 3 Laboratory of Molecular Physiology, HSE University, Moscow, Russia; University of Maryland School of Medicine, Baltimore, Maryland, USA

**Keywords:** *Aspergillus*, peptidases, protein C

## Abstract

*Aspergillus ochraceus* VKM-F4104D (L-1) is a saprotrophic fungus isolated from buried soils of Phanagoria (Russia). This strain is known as a producer of the fibrinolytic peptidase-activating plasma protein C. We have sequenced and assembled its genome for a more detailed understanding of the fungus’ physiology and encoded peptidases.

## ANNOUNCEMENT

*Aspergillus ochraceus* VKM-F4104D (L-1) is a known producer of a peptidase possessing the unique property of activating protein C of human blood plasma (1). It was previously identified by ITS sequencing (available at GenBank under the accession number KC859009) and deposited in the Russian collection of industrially important microorganisms (“VKPM”) (2). Initially, *A. ochraceus* was isolated on malt-extract agar (MEA) by a serial dilution method from samples of buried soils during excavations of Phanagoria, the largest ancient Greek city on the Taman peninsula (Russia, 44.47035, 38.97919).

*A. ochraceus* VKM-F4104D was cultivated on MEA slants at 28°C. After 7 days, it was used to prepare a spore suspension, which was then inoculated into 100 mL of the liquid medium (g/L: malt extract—67.0, glucose—20.0, and peptone—1.0) and cultivated for 48 hours at 28°C, 200 rpm (3). The biomass was subsequently harvested by centrifugation and used for DNA isolation.

The genomic DNA was isolated using GNOME DNA Isolation Kit (MP Biomedicals). Libraries were prepared with KAPA HyperPrep Kit (Roche) and sequenced on Illumina NextSeq with 2 × 150 bp reads. A total of 41,018,432 reads were generated. Quality control of raw reads was performed using FastQC 0.12.1 (4) and SPAdes 3.15.5 (5) was used to assemble reads into contigs. Assembly quality was evaluated with QUAST 5.2.0 (6). Genes were predicted with AUGUSTUS 3.5.0 using --species=aspergillus_fumigatus (*Aspergillus fumigatus*) and --genemodel=complete options (7). For other tools, the default parameters were used.

The assembled genome consists of 862 contigs longer than 200 bp, with a maximum contig length of 720,519 bp. The total length is 36,493,367 bp. The assembled genome is available in the NCBI database with the accession number JBAFXH000000000 and raw reads with SRR28732222. The GC content of the genome is 49.2%. *N*50 and *L*50 values equal 131 kb and 89, respectively. The average coverage depth for the assembled genome is 319x. A total of 11,038 putative proteins were predicted to be encoded on the genome.

**Fig 1:**
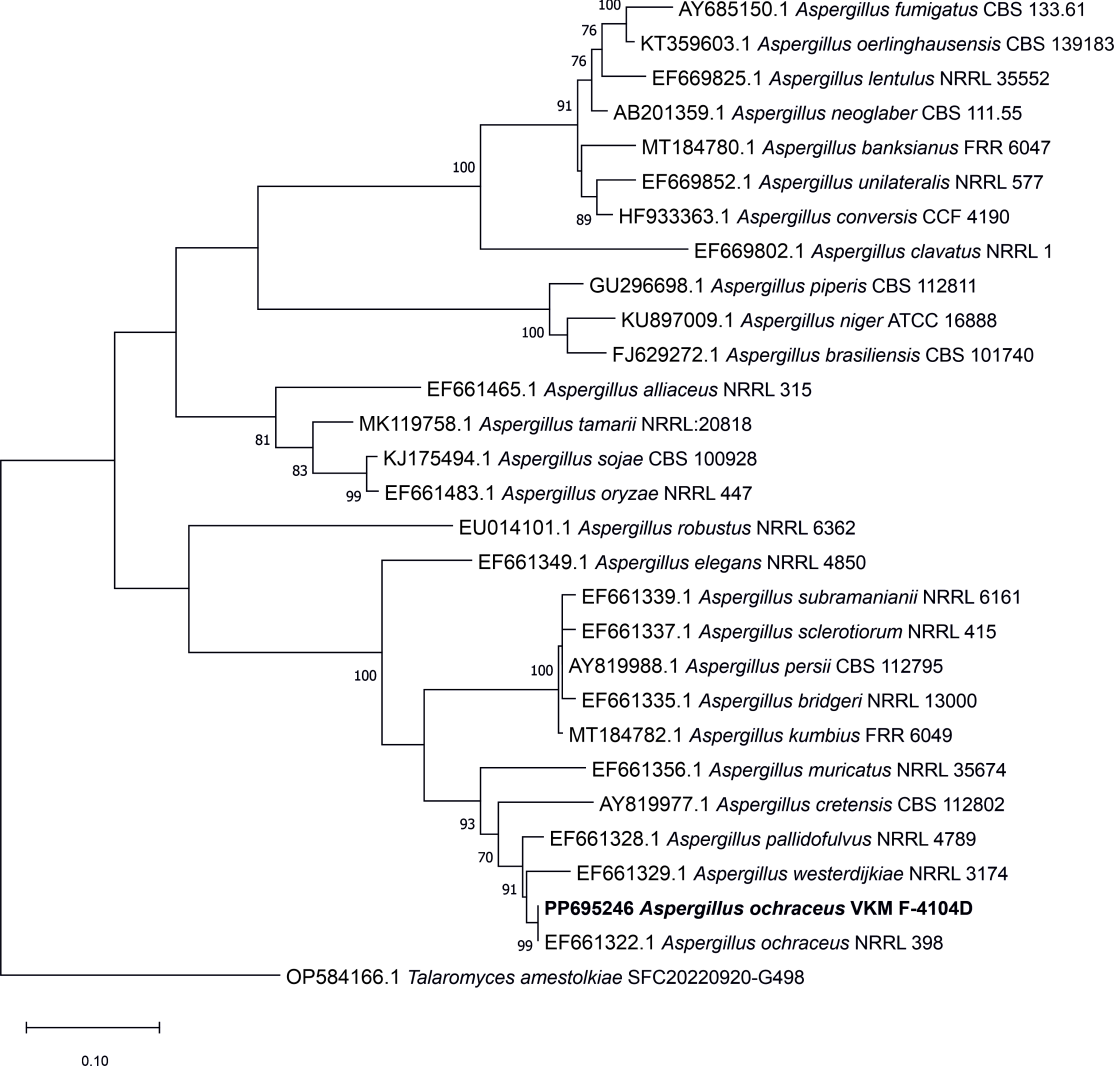
Phylogenetic tree illustrating the taxonomy of *A. ochraceus* VKM-F4104D based on the *BenA* coding sequence.

A maximum-likelihood phylogenetic tree based on the alignment of 18 beta-tubulin coding sequences [*BenA, in silico* amplified using Pincer (8) and Bt2a and Bt2b primers (9)] done with muscle (version 3.8.31) (10) was constructed in MEGA 11 with automated model selection, confirming that strain VKM-F4104D is indeed *A. ochraceus* (Fig. 1).

## Data Availability

The assembled genome was deposited at GenBank under the accession number JBAFXH00000000 and the BioProject accession number PRJNA1075430. The raw sequence reads have been submitted to the Sequence Read Archive (SRA) under the accession number SRR28732222. ITS sequence previously used for identification is available at GenBank under the accession number KC859009. mRNA sequence of the BenA is available under the accession number PP695246.
